# Effect of nano-graphene lubricating oil on particulate matter of a diesel engine

**DOI:** 10.1038/s41598-024-61694-z

**Published:** 2024-05-11

**Authors:** Xin Kuang, Xiping Yang, Hao Fu, Shengyong Li, Hua Bian

**Affiliations:** 1 School of Traffic Engineering, Jiangsu Shipping College, Nantong, 226010 China; 2 School of Intelligent Manufacturing and Information, Jiangsu Shipping College, Nantong, 226010 China; 3 School of Mechanical Engineering, Jiangsu University, Zhenjiang, 212013 China

**Keywords:** Nano-graphene, Lubricating oil, Particulate matter, Physicochemical properties, Mechanical engineering, Graphene, Nanoparticles, Environmental impact, Energy conservation, Environmental impact

## Abstract

Nano-graphene lubricating oil with appropriate concentration shows excellent performance in reducing friction and wear under different working conditions of diesel engines, and has been widely concerned. Lubricating oil has a significant impact on particulate matter (PM) emissions. At present, there are few studies on the impact of nano-graphene lubricating oil on the physicochemical properties of PM. In order to comprehensively evaluate the impact of nano-graphene lubricating oil on diesel engines, this paper mainly focused on the effects of lubricating oil nano-graphene additives on the particle size distribution and physicochemical properties of PM. The results show that, compared with pure lubricating oil, the total number of nuclear PM and accumulated PM of nano-graphene lubricating oil is significantly increased. The fractal dimension of PM of nano-graphene lubricating oil increases and its structure becomes more compact. The average fringe separation distance of basic carbon particles decreases, the average fringe length increases. The degree of ordering and graphitization of basic carbon particles are higher. The fringe tortuosity of basic carbon particles decreases, and the fluctuation of carbon layer structure of basic carbon particles decreases. Aliphatic substances in PM are basically unchanged, aromatic components and oxygen functional groups increase. The initial PM oxidation temperature and burnout temperature increase, the maximum oxidation rate temperature and combustion characteristic index decrease, and the activation energy increases, making it more difficult to oxidize. This was mainly caused by the higher graphitization degree of PM of nano-graphene lubricating oil and the increased content of aromatic substances.

## Introduction

Lubricating oil is known as the “blood” of the engine, which is one of the key factors affecting the mechanical efficiency and reliability of the engine. With the implementation of National VI emission standards^1^, and under the pressure of energy shortage and harsh working conditions, the quality requirements of engine lubricating oil become more stringent, especially the excellent anti-friction and wear-resisting properties^2,3^. With the development of nanotechnology, more and more researchers study lubricating oil nano-additives with anti-friction and wear-resisting characteristics, such as soft metals^4,5^, metal compounds^6–9^, organic compounds^10,11^, graphite and its interlayer compounds^12–15^, etc. Among them, two-dimensional nano-graphene is more attention^12,16,17^. A large number of studies^18–23^ have shown that the tribological properties of general industrial lubricanting oil can be improved by adding nano-graphene to them.

In recent years, researchers began to study the addition of nano-graphene into engine lubricating oil, focusing on dispersion stability and tribological properties. Paul et al.^24^ studied the dispersion stability and tribological properties of dodecylamine functionalized graphene lubricating oil. The results showed that the dispersion stability of the functionalized graphene lubricating oil was guaranteed and a friction film was formed on the surface of the friction pair. Compared with the engine basic lubricating oil, the friction coefficient of the graphene lubricating oil was reduced by 40%, and the anti-wear performance was improved. Ali et al.^25^ tested the tribological and wear properties of nano-graphene lubricating oil based on ASTMG181 standard and connected it with actual engine performance. The results showed that the tribological properties and anti-wear properties of the nano-graphene lubricating oil were improved by 29–35% and 22–29% respectively, and the simulated fuel consumption under road load in the new European driving cycle test was reduced by 17%. Guimarey et al.^26^ prepared nano-graphene by electrochemical stripping method and studied the tribological properties of nano-graphene lubricating oil. The research showed that nano-graphene lubricating oil with 0.05% mass fraction had better tribological properties. Kuang et al.^27^ used oleic acid and stearic acid to chemically modify nano-graphene, and studied the tribological properties of the cylinder liner and piston specimen, a key friction pair of the engine, under the action of nano-graphene lubricating oil. The results showed that the dispersion stability of the modified nano-graphene lubricating oil was improved, and the addition of 25 ppm mass fraction nano-graphene in the lubricating oil had the best friction and wear reduction effect.

Nano-graphene lubricating oil with appropriate concentration shows excellent performance in reducing friction and wear. Different from general purpose industrial lubricants, engines are currently required to transition to Euro VI, national VI and above ultra-low emission regulations. Therefore, it is not only necessary to consider the dispersion stability and the effect of friction and wear reduction under engine working conditions, but also to consider the impact of engine oil consumption on emissions, especially PM emissions^28,29^.

Ali et al.^25^ added nano-graphene with a sheet diameter of 5–10 μm and a thickness of 3–10 nm to Castrol 5W-30 lubricating oil at a mass concentration of 0.4%. The New European Driving Cycle (NEDC) test of nano-graphene lubricating oil and pure lubricating oil was carried out respectively, and the emission characteristics of the engine under different speeds and loads were compared under the same conditions. The results showed that under most working conditions, the emission performance of nano-graphene lubricating oil was improved, CO_2_ emission was reduced by 3.4–4.66%, NO_X_ was reduced by about 3–5%, and HC decreased with the increase of engine load. This shows that the addition of nano-graphene will have a certain impact on CO_2_, HC, NO_X_ emissions, but there is a lack of attention to PM emissions. Marcano et al. investigated the effect of lubricating oil with nano-MoS_2_ on a diesel engine from a practical application point of view. The results showed that the ultrafine PM produced by the addition of nano-powders increased, and HC and CO emissions increased^30^.

In view of the influence of nano-graphene additives on emissions, the existing literature has preliminarily studied the influence of nano-graphene lubricating oil on emissions of CO, CO_2_, HC, NO_X_ and PM. However, the effect of nano-graphene lubricating oil on the physicochemical properties of PM, the formation mechanism of PM and its potential impact on the post-treatment system are more noteworthy. Therefore, in this paper, nano-graphene was modified and then added into lubricating oil to further study the effects of nano-graphene additives on particle size distribution, microscopic morphology and structure, degree of graphitization, surface functional groups and oxidation characteristics of PM, which provided an important reference for the practical application of nano-graphene in engine lubricating oil.

## Test device and method

This paper mainly focused on the study of the effects of nano-graphene lubricating oil on particle size distribution and physicochemical properties of PM. Considering the time and cost of the study, the single-cylinder diesel engine, KD192FW, was selected for bench test. The relevant technical parameters are shown in Table 1. And by adding lubricating oil into diesel and burning, the effect of nano-lubricating oil on particle size distribution was measured online, and appropriate amount of PM was collected for characterization, so as to study the action mechanism of nano-lubricating oil on physicochemical properties of PM.

**Table 1:** KD192FW diesel engine technical parameters.

Parameter	Index
Engine type	Single-cylinder, four-stroke, air-cooled, vertical type
Piston stroke	75 mm
Total displacement	0.499 L
Rated power/speed	7.6 kW/3000 r·min^−1^
Maximum torque/speed	25 N·m/2500 r·min^−1^
Compression ratio	19.5

### Test materials

Commercially available 0# National 5 diesel was selected for the test, and its physicochemical properties are shown in Table 2.

**Table 2:** Physicochemical properties of diesel oil used in test.

Parameter	Index
Density (20 °C, kg/m^3^)	836
Boiling point (°C)	57
Lower heating value (MJ/kg)	42.90
Ash content (%)	0.001
Cetane number	51.5
Kinematic viscosity (20 °C, mm^2^/s)	5.055

**Figure 1:**
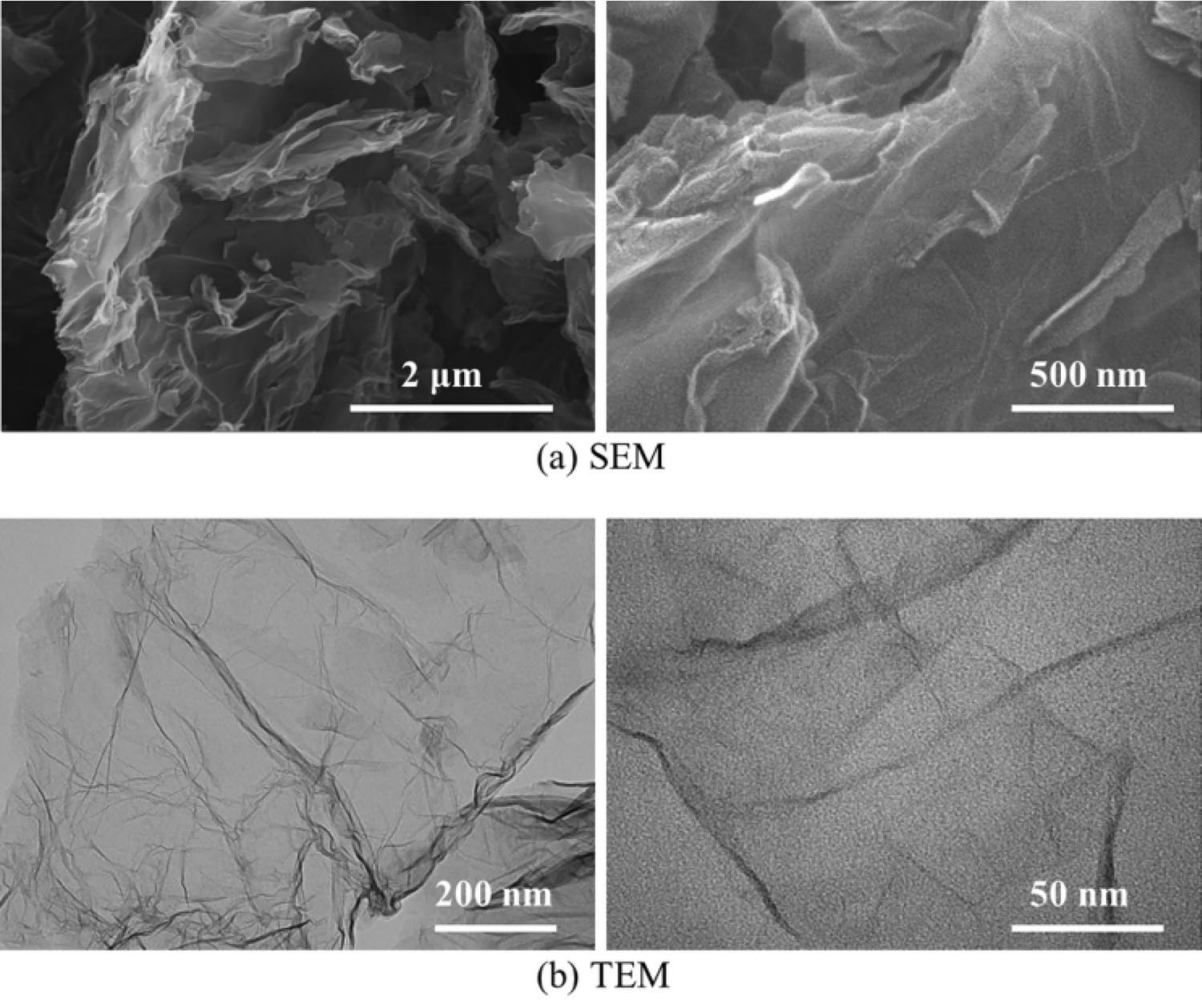
SEM and TEM image of nano-graphene.

Nano-graphene was commercially available, and its electron microscopic images are shown in Fig. 1. Through the measurement of Digital Micrograph software, it can be seen that the average thickness of nano-graphene is about 0.5–1 nm, and the number of nano-graphene layers is about 1–3. Nano-graphene was chemically modified by oleic acid and stearic acid in the following ways^27^. Firstly, 0.5 g of nano-graphene was dispersed into 100 mL anhydrous ethanol. Then 2 g stearic acid and 3 g oleic acid were added to the mixture. Finally, the oil soluble graphene was obtained by centrifugal drying after being stirred for 4 h at 80 °C. Finally, the oil soluble graphene was obtained by centrifugal drying after being stirred for 4 h at 80 °C. Previous experimental results^27^ have shown that the dispersion stability of the modified graphene lubricating oil was improved, and the modified graphene lubricating oil with 25 ppm concentration had the best tribological properties. The modified nano-graphene with a mass concentration of 25 ppm was weighed into the lubricating oil by a precision balance. After the strong stirring of the magnetic mixer and the action of the high-frequency ultrasonic disperser, it was intermittently dispersed at low temperature (25 ± 2 °C) until it was stably dispersed in the lubricating oil to obtain the nano-graphene lubricating oil, which was referred to as MGL25. Pure lubricating oil (5W-30 SN/CF) was denoted as PLO. The lubricating oil used in the test was PLO and MGL25 respectively.

**Figure 2:**
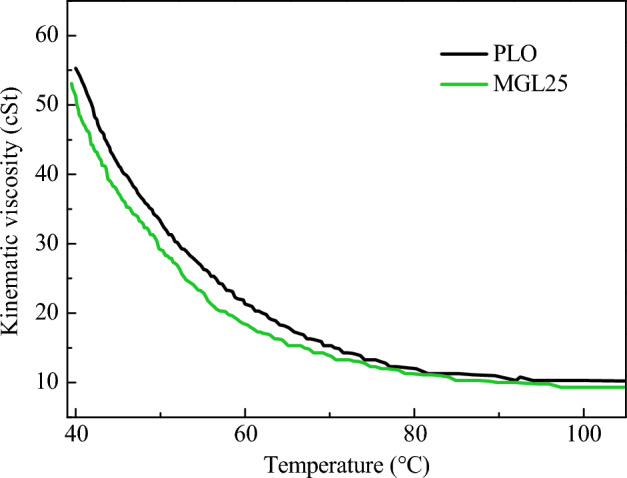
Change of kinematic viscosity of different lubricating oil with temperature.

In this paper, the kinematic viscosity of lubricating oil at 40–100 °C was measured by reference to the standard GB/T 265–1988^31^, kinematic viscosity measurement method and dynamic viscosity calculation method of petroleum products. The kinematic viscosity changes of PLO and MGL25 with temperature are shown in Fig. 2. It can be seen that the kinematic viscosity of MGL25 decreases compared with PLO. This is because graphene slides between layers to form self-lubrication, and the appropriate amount of graphene added into the lubricating oil is conducive to reducing the internal friction when the lubricating fluid flows.

In 2003, China issued the standard “Automotive Engine Reliability Test Method” GB/T19055-2003^32^, which clearly stipulates that the ratio of lubricating oil to fuel consumption at full load and rated speed shall not exceed 0.3%. In order to accelerate the generation and influence of lubricating oil on PM, the fuel in the test was two kinds of mixed fuel, namely PLO with 0.5% mass fraction added in diesel and MGL25 with 0.5% mass fraction added in diesel. The lubricating oil in the oil pan is the same as the lubricating oil added to the diesel. The lubricating oil and diesel are easy to be miscible, and the mixed fuel can be obtained by fully stirring with a stirring rod.

### PM online measurement equipment and scheme

#### Test equipment and instruments

**Figure 3:**
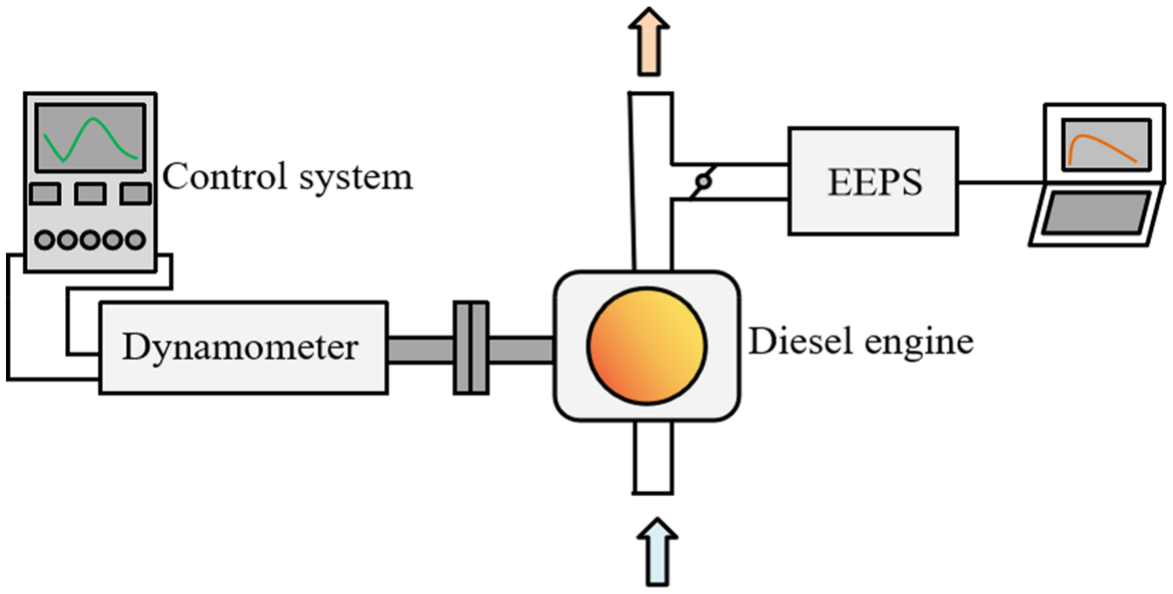
Test equipment for on-line measurement of particle size distribution.

The schematic diagram of the test equipment for online measurement of particle size distribution is shown in Fig. 3. The test bench mainly includes diesel engine, dynamometer, control system, particle size spectrometer, etc. The test instruments and equipment are shown in Table 3. During the test, the diesel engine was mounted on the AC electric dynamometer, and the control of the diesel engine start-stop and operation conditions was completed by the control system EST2010. An American 3090 particle size spectrometer (EEPS) was used in the test, with a sampling flow of 50 L/min.

**Table 3:** Main instruments and equipment for testing.

Instrument	Model number	Producer
Dynamometer	CWAC-YVP-15-50/2	Taiyuan Zhongcheng
Control system	EST2010	Hangzhou Zhongcheng
Particle size spectrometer	EEPS 3090	American TSI

#### Test scheme

Before the test, the condition of the test bench was checked. After the oil, water and air routes were normal, opened the bench test control interface and started the engine. In order to ensure the reliability of the test data, the test data should be collected at the ambient temperature of 25 ± 3 °C, humidity of 55 ± 3%, cooling water temperature of 80 ± 3 °C, and fuel temperature of 30 ± 3 °C. After the engine idled for half an hour, the formal test began when the lubricating oil temperature reached about 80 ± 3 °C. At the same time, the particle size spectrometer was opened and preheated for about half an hour. In order to study the effect of nano-graphene lubricating oil on particle size distribution, a comparative test was conducted. The fuel and lubricating oil used in the test are shown in Table 4. The position about 10 cm away from the exhaust pipe was used as the sampling point of EEPS3090 particle size spectrometer. At this point, a stainless steel pipe with a diameter of 6 mm was used to connect the exhaust pipe. The pipe wall is smooth without bending, so as to minimize the resistance to PM. Specific test operating parameters are shown in Table 5 below. At each operating point, after the engine ran stably for 5 min, EEPS was used for sampling measurement at the sampling point. For all tests, particle size distribution measurement was taken thrice, each time with an interval of 30 s, to guarantee the reproducibility of the results.

**Table 4:** Test oil.

Test number	Fuel	Lubricating oil
1	Diesel fuel mixed with 0.5% PLO	PLO
2	Diesel fuel mixed with 0.5% MGL25	MGL25

**Table 5:** Specific test operating parameters.

Speed (r/min)	Load (% of full load)
3000	10, 25, 50, 75, 100
2000, 2250, 2500, 2750, 3000	100

Different fuels and lubricants were tested in turn. After the completion of an oil test, the fuel in the fuel pipe was discharged, and the fuel pipe was cleaned with the fuel to be tested. The engine ran stably for an additional 20 min to ensure that the entire oil circuit was filled with the test fuel to avoid the interference of the previous fuel on the test results. Moreover, the lubricating oil added to the fuel was consistent with the lubricating oil in the oil pan. Each time one kind of lubricating oil was replaced, the original lubricating oil in the engine oil pan was discharged and fresh test lubricating oil was added. Run the engine until the water temperature and lubricating oil temperature reached the appropriate value. After running for half an hour at a 215 speed of 3000 r/min and a torque of 24.2 N·m, the lubricating oil in the engine was discharged to clean the non-test lubricating oil in the engine, and the new lubricating oil used in the test was added again. And the same method was used to ensure that the fuel and lubricating oil used in the first test were new oil.

### PM collection device and scheme

#### Test equipment and instruments

**Figure 4:**
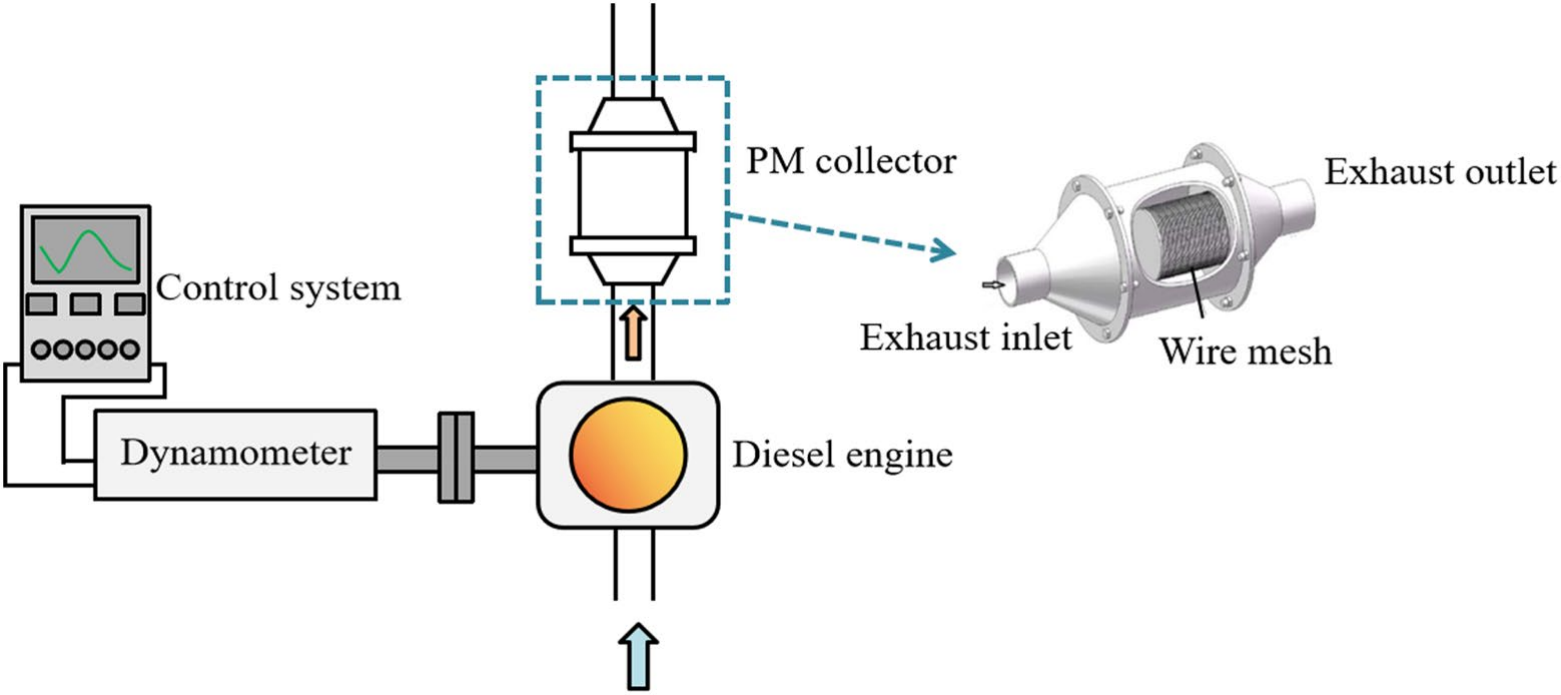
Schematic diagram of test device for PM collection.

In order to study the effects of nano-lubricating oil on the physicochemical properties of PM, PM collection and comparative tests were carried out. A self-made sampling device with wire mesh was used to collect PM samples. The schematic diagram of the test equipment for PM collection is shown in Fig. 4. The fuel and lubricating oil used in the test are shown in Table 4. In each test, the machine was shut down after stable operation for 90 min at the calibration operating point, and the PM adsorbed in the particle sampling device were scraped down and stored in clean glassware. In order to replace oil products infrequently and ensure the reliability of data, for each type of lubricating oil, PM collection was carried out immediately after the particle size distribution test, and the lubricating oil was replaced after the PM collection was completed.

#### Test scheme

The microscopic morphology, structure, graphitization degree, surface functional groups and oxidation characteristics of the collected PM were studied by using relevant characterization instruments. Among them, the graphitization degree of PM was measured by Raman DXR spectrometer. The sample preparation method was as follows. Appropriate amount of nano-graphene powder was put on a clean transparent slide, which was compacted and placed on the sample table for testing. The surface functional groups of PM were measured by Fourier infrared spectrometer Nicolet iS-50. The sample preparation method was as follows. Appropriate amount of nano-graphene powder and dried potassium bromide were mixed and ground in an agate bowl, then the mixture was put into a solid tablet mold and kept under proper pressure for 2 min to obtain translucent ingotted pieces, which were loaded into a test rack for detection. The morphologies and crystal structures of basic carbon particles were photographed by JEM-2100(HR) field emission transmission electron microscope (TEM). The point resolution of the instrument can reach 0.23 nm, the line resolution can reach 0.14 nm, and the magnification error is less than or equal to ± 10%. Particle samples need to be pretreated before testing. The treatment method was as follows. A small amount of PM were placed in anhydrous ethanol, ultrasonic shock was carried out for 15 min, and after standing for 5 min, a small amount of the upper layer solution was dropped on the copper grid microgrid by pipette. After drying, it was put into the sample table of high-power TEM for measurement. More than 20 TEM images were taken in each case. Digital Micrograph software was used to accurately and quantitatively analyze the microstructure parameters of basic carbon particles in the high-power TEM image, and analyze the influence of different nano-graphene lubricating oil on the microstructure of basic carbon particles. The oxidation characteristics of PM were measured by Swiss TGA/DSC1 thermogravimetric analyzer. The test conditions were as follows. High purity nitrogen was selected as the protection gas, N_2_ (80%) and O_2_ (20%) were selected as the reaction gas, and the gas flow rate was 50 mL/min. The initial temperature was room temperature, the heating rate was 10 °C/min after heating to 40 °C, and the termination temperature was 800 °C. A ceramic crucible with high temperature resistance was selected for the sample pool. The temperature accuracy of the instrument is ± 0.2 °C, the weight range is 0–1 g, the balance sensitivity is 0.01 μg. Before the formal experiment, two sets of blank tests were carried out and the background was deducted to reduce the test error. The thermogravimetric (TG) curve was obtained from the test results. The first derivative of the TG curve was calculated to obtain the curve of the sample mass loss rate changing with temperature, which was the thermogravimetric derivative (DTG) curve. Thermogravimetric analysis parameters of different lubricating oils were calculated according to TG and DTG curves to analyze the evaporative oxidation characteristics and thermal stability of lubricating oils.

#### Morphology and structure of PM

The shape and structure of PM produced by diesel engine are irregular and complex^33–35^. The fractal dimension D_f_ was proposed to describe the degree of density and the degree of geometric structure irregularity among the basic carbon particles of PM^36–40^.

The D_f_ of agglomerated particles can be obtained by the calculation formula (1) after extracting the corresponding parameters from the TEM image.

1 $$N={{{\text{k}}}_{{\text{g}}}(\frac{{R}_{g}}{{r}_{p}})}^{{D}_{f}}$$

In the formula (1), k_g_ is the structural coefficient, which is related to the radius of rotation and r_p_ is the average diameter of the basic carbon particles. Logarithmic calculation of both sides of the formula gives the following formula (2):

2 $$lgN={{\text{lgk}}}_{{\text{g}}}+{D}_{f}{\text{lg}}({R}_{g}/{r}_{p})$$

In formula (1), lg(R_g_/r_p_), lgN are respectively as a variable, x, y coordinates. lgk_g_ and D_f_ are constant. lgN and lg(R_g_/r_p_) is a linear correlation. The linear slope obtained by fitting lgN−lg(R_g_/r_p_) curve is the D_f_ of particles. In the formula, R_g_ is the gyration radius of aggregated particles, which can be derived from the following formula (3).

3 $${ R}_{g}=\sqrt{\frac{1}{N}\sum_{i=1}^{N}{{r}_{i}}^{2}}$$

In the formula (3), r_i_ is the distance between the center of mass of aggregated particles and the center of mass of a single basic carbon particle. The mass center point of PM cannot be obtained in actual TEM image processing, and R_g_ is difficult to be accurately measured. At the same time, there is superposition of PM in TEM images, which makes it difficult to calculate N. So R_g_ and N have to be derived indirectly. R_g_ can be calculated according to Brasil algorithm:

4 $$\frac{L}{2{R}_{g}}=1.5\pm 0.05$$

In the formula, L is the maximum projected length of aggregated PM.

N is the number of basic carbon particles of PM, which can be obtained by the following formula (5) according to the projected area of aggregated PM.

5 $$N={{{\text{k}}}_{{\text{a}}}(\frac{{A}_{a}}{{A}_{p}})}^{{{\text{a}}}_{{\text{a}}}}$$

In the formula, ${A}_{a}$ is the projected area of aggregated PM, ${A}_{p}$ is the average projected area of basic carbon particles, and ${{\text{k}}}_{{\text{a}}}$ and ${{\text{a}}}_{{\text{a}}}$ are empirical constants. According to the general empirical data, ${{\text{k}}}_{{\text{a}}}$ is 1.81 and ${{\text{a}}}_{{\text{a}}}$ is 1.19^41^.

**Figure 5:**
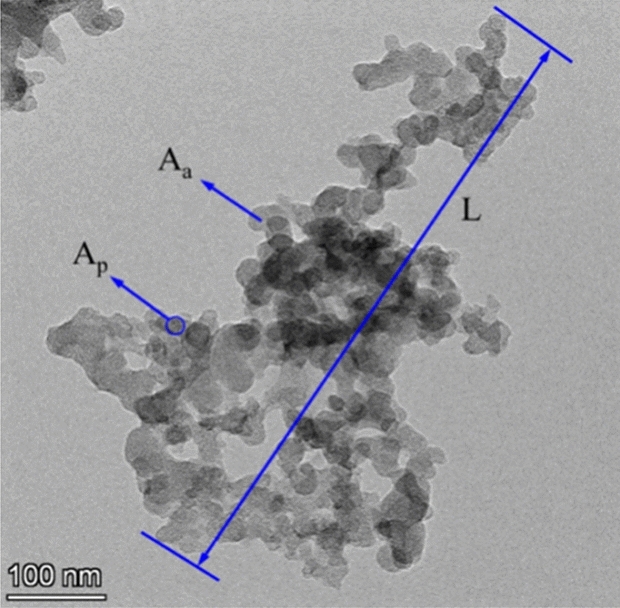
Schematic diagram of typical PM morphology parameters.

The parameters *r*_*p*_, ${A}_{a}$, ${A}_{p}$ and L can be obtained by processing and analyzing TEM images with Digital Micrograph software, and the D_f_ of PM can be calculated. Typical PM morphology parameters are shown in Fig. 5. In this study, relevant parameters of 10–20 aggregated particles under the rated condition were measured for statistical purposes statistics.

#### Oxidation characteristics of PM

In order to describe and compare the oxidation process of different particles, three specific temperature points in the oxidation process were selected as characteristic parameters, including maximum oxidation rate temperature T_max_, initial oxidation temperature T_i_ and burnout temperature T_h_.

In order to further evaluate the oxidation characteristics of PM, combustion characteristic index S was used to compare the combustion conditions of PM corresponding to different lubricating oil. The calculation formula (6) is as follows:

6 $${\text{S}}=\frac{{(\frac{dw}{dt})}_{{\text{max}}}{(\frac{dw}{dt})}_{{\text{mean}}}}{{{\text{T}}}_{{\text{i}}}^{2}{{\text{T}}}_{{\text{h}}}}$$

In the formula, ${(\frac{dw}{dt})}_{{\text{mean}}}$ is the average combustion velocity and ${(\frac{dw}{dt})}_{{\text{max}}}$ is the maximum combustion velocity^42^.

According to the Arrhenius theorem, the oxidation rate of PM is directly related to the apparent activation energy of the reaction, E_α_. The kinetic Eq. (7) of non-uniform phase system under non-isothermal condition is shown as follows:

7 $$\frac{d\alpha }{dT}=\frac{{\text{A}}}{\upbeta }{{\text{e}}}^{-\frac{{{\text{E}}}_{\mathrm{\alpha }}}{{\text{R}}T}}f(\alpha )$$

In the formula, α is the mass loss rate of PM, %; T is the thermodynamic temperature, K; R is the gas constant, and its value is 8.314 J/(mol·K). A is the pre-exponential factor; β is the heating rate, K/min; E_α_ is the apparent activation energy, J/mol; f(α) is the combustion kinetic mechanism function.

In this study, the Coats-Redfem integral method was used to calculate the dynamic parameters of PM. The multistage reaction function f(α) of PM and oxygen can be expressed as f(α) = (1−α)^n^, which is brought into the formula.

8 $$\frac{d\alpha }{dT}=\frac{{\text{A}}}{\upbeta }{e}^{-\frac{{{\text{E}}}_{\mathrm{\alpha }}}{{\text{R}}T}}{(1-\alpha )}^{n}$$

Taking the logarithm after integrating both sides of formula (8), we get:

9 $$ {\text{ln}}\left[ {\frac{{1 - ln\left( {1 - \alpha } \right)^{1 - n} }}{{T^{2} \left( {1 - n} \right)}}} \right] = {\text{ln}}\left[ {\frac{{{\text{AR}}}}{{{\beta E}_{{\upalpha }} }}\left( {1 - \frac{{2{\text{R}}T}}{{{\text{E}}_{{\upalpha }} }}} \right)} \right] - \frac{{{\text{E}}_{{\upalpha }} }}{{{\text{R}}T}}\left( {n \ne 1} \right) $$

10 $$ {\text{ln}}\left[ {\frac{{ - {\text{ln}}\left( {1 - \alpha } \right)}}{{T^{2} }}} \right] = {\text{ln}}\left[ {\frac{{{\text{AR}}}}{{{\beta E}_{{\upalpha }} }}\left( {1 - \frac{{2{\text{R}}T}}{{{\text{E}}_{{\upalpha }} }}} \right)} \right] - \frac{{{\text{E}}_{{\upalpha }} }}{{{\text{R}}T}}\left( {n = 1} \right) $$

For the characteristics of the reaction temperature zone and activation energy E_α_ in the conventional thermogravimetric test of PM, $\frac{2{\text{R}}T}{{{\text{E}}}_{\mathrm{\alpha }}}$ is much less than 1. Therefore, the first term on the right side of the formula (9) and formula (10) can be simplified as ln $\frac{{\text{AR}}}{\upbeta {{\text{E}}}_{\mathrm{\alpha }}}$, which is a constant.

According to the research of relevant scholars, the oxidation reaction order of diesel engine PM is approximately 1. Therefore, formula (10) is simplified as follows:

11 $$ {\text{ln}}\left[ {\frac{{ - {\text{ln}}\left( {1 - \alpha } \right)}}{{T^{2} }}} \right] = {\text{ln}}\left[ {\frac{{{\text{AR}}}}{{{\beta E}_{{\upalpha }} }} - \frac{{{\text{E}}_{{\upalpha }} }}{{{\text{R}}T}}} \right] $$

This formula can be seen as a straight line, in which 1/T is as variables, $-\frac{{{\text{E}}}_{\mathrm{\alpha }}}{{\text{R}}}$ is as slope, ln $\frac{{\text{AR}}}{\upbeta {{\text{E}}}_{\mathrm{\alpha }}}$ is as intercept, and ln[$\frac{-{\text{ln}}(1-\alpha )}{{T}^{2}}$] is as dependent variables. E_α_ can be obtained by calculating the slope of the line by linear fitting method. Then, E_α_ is substituted into formula (11) to solve the pre-exponential factor A.^43^.

## Results and discussion

### Particle size distribution

The particle size distribution results under different torques and speeds were obtained through the test. In the test results, the quantity concentration of PM is expressed in the form of dN/dlogD_p_(/cm^3^), where N was the number of PM and D_p_ was the PM size. The quantity concentration and particle size of PM are related to the running condition of diesel engine, and the logarithmic form is advantageous for comparison. After statistical analysis of the test data, it is found that there were almost no particles with particle size between 250 and 560 nm, so the figure only shows particle size distribution between 5.6 and 250 nm.

#### Particle size distribution at rated speed

**Figure 6:**
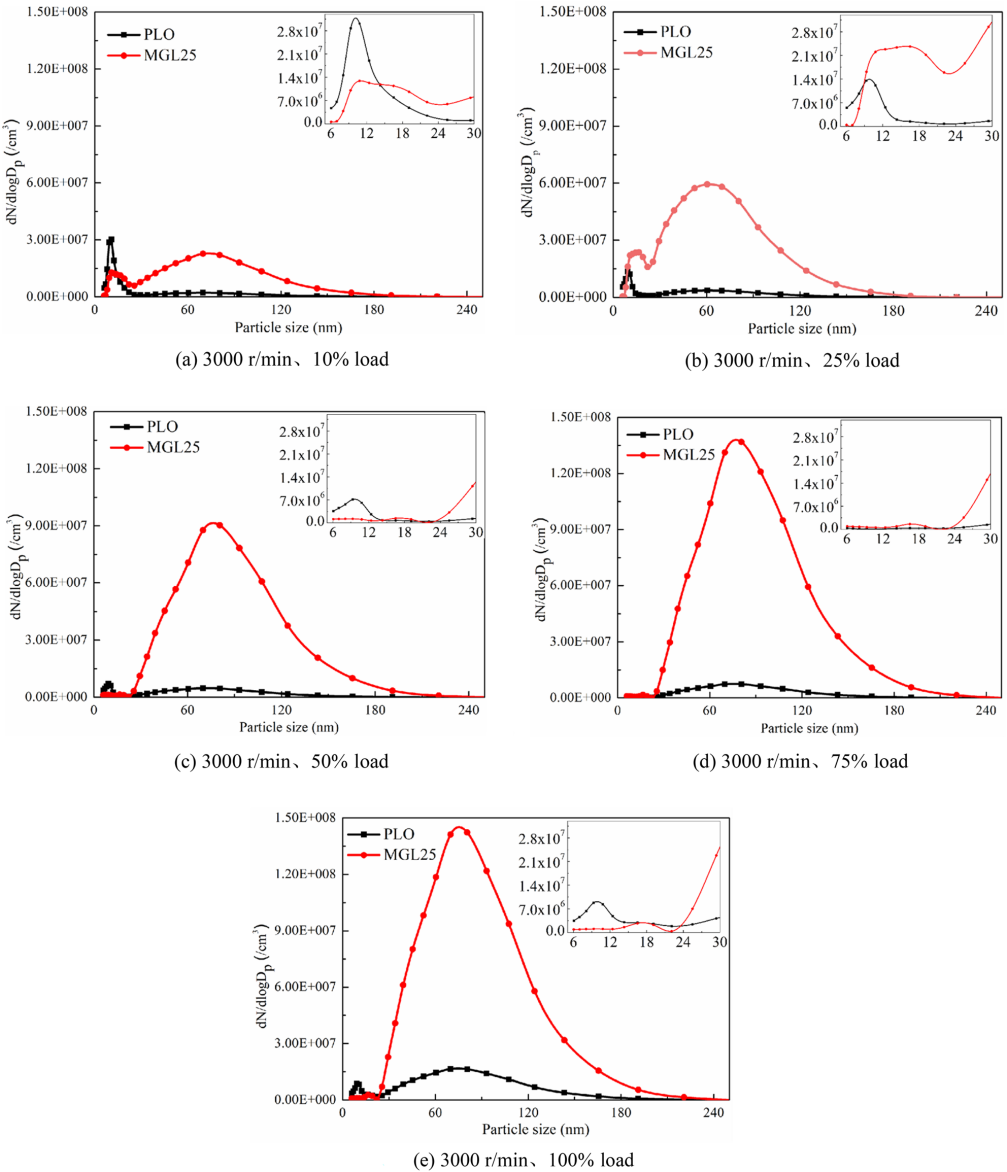
Particle size distribution of different lubricating oils.

Figure 6 respectively shows the changes in particle size distribution of different lubricating oils added to the fuel under different load conditions at the rated speed of 3000 r/min. It can be clearly seen from the figure that the particle size all presents a bimodal logarithmic distribution, with the peaks occurring at 9–19 nm and 69–81 nm, which is basically consistent with the peak position in the literature^44^. The quantity concentration of accumulated particles of MGL25 is significantly higher than that of PLO, and this phenomenon is more obvious at large loads. In the enlarged diagram of the distribution interval with particle size below 30 nm, it can be seen that the corresponding peak value tends to decrease with the increase of load. Compared with PLO, the peak value of particle size corresponding to MGL25 migrated to a larger particle size range. The peak number of particles in the 60–80 nm particle size range corresponding to MGL25 reached 10^7^–10^8^, which is the same order of magnitude as in the literature^45^. It’s much higher than that of PLO. This is due to the existence of nano-graphene, which is easier to self-nucleate, and more easily adsorbed on the carbon surface or agglomerate, forming larger particle size particles. And the larger the load, the higher the temperature in the combustion chamber, the more likely to agglomerate, producing larger particle size particles.

**Figure 7:**
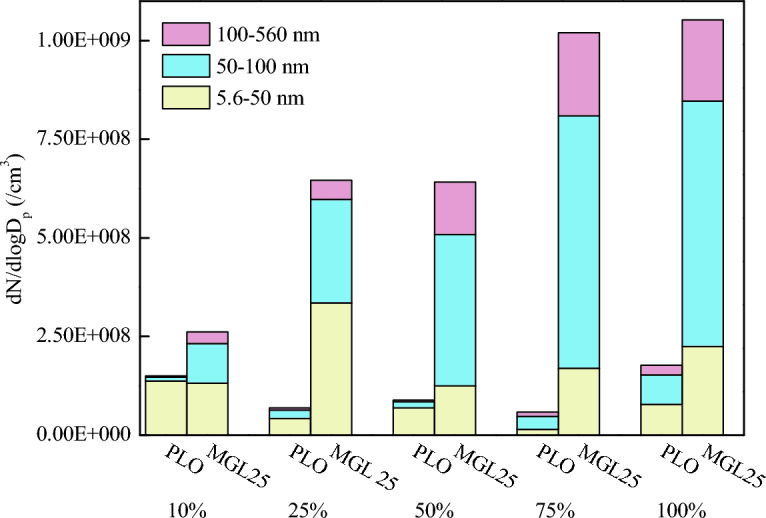
Particle quantity concentration in three particle size intervals corresponding to two lubricating oils at rated speed and different loads.

The particle size corresponding to the two lubricating oils at the rated speed and different loads was counted according to three intervals of 5.6–50 nm, 50–100 nm and 100–560 nm. The statistical results are shown in Fig. 7. As can be seen from the figure, the particle size is mostly concentrated below 100 nm that is, the total height of the white and green parts, which is similar to the results in the reference^44^.

For PLO, compared with other particle size intervals, the number of nuclear particles in the particle size range of 5.6–50 nm is the largest. The number of nuclear particles plays a leading role in the total number of particles. With the increase of load, the quantity concentration of accumulated particles tends to increase. For nano-graphene lubricating oil, with the increase of load, the quantity concentration of accumulated particles shows an increasing trend. The amount of PM at 5.6–50 nm, 50–100 nm and 100–560 nm corresponding to nano-graphene lubricating oil is greater than that corresponding to PLO. The amount of PM in the accumulated state increases more significantly than that in the nuclear state. Studies have shown that PM with a particle size of less than 100 nm can pass through the alveoli and enter the blood, which is very harmful to human health. This means that the disadvantage of nano-graphene lubricating oil is more prominent. If nano-graphene lubricating oil is used, it is necessary to adjust the control strategy and test and calibrate the post-processing system of the engine to reduce the discharge of ultrafine particles into the atmosphere. This is because when the nano-graphene lubricating oil is involved in combustion, the nano-graphene particles may self-nucleate, resulting in the risk of increasing nuclear particles. These self-nucleating particles and their aggregates will be directly adsorbed on the carbon surface or further agglomerated with carbon particles to form accumulated particles, resulting in the risk of increasing aggregated particles. In addition, with the increase of load, the temperature increases, resulting in the increase of agglomeration and accumulated particles.

#### Particle size distribution under 100% load at different speeds

**Figure 8:**
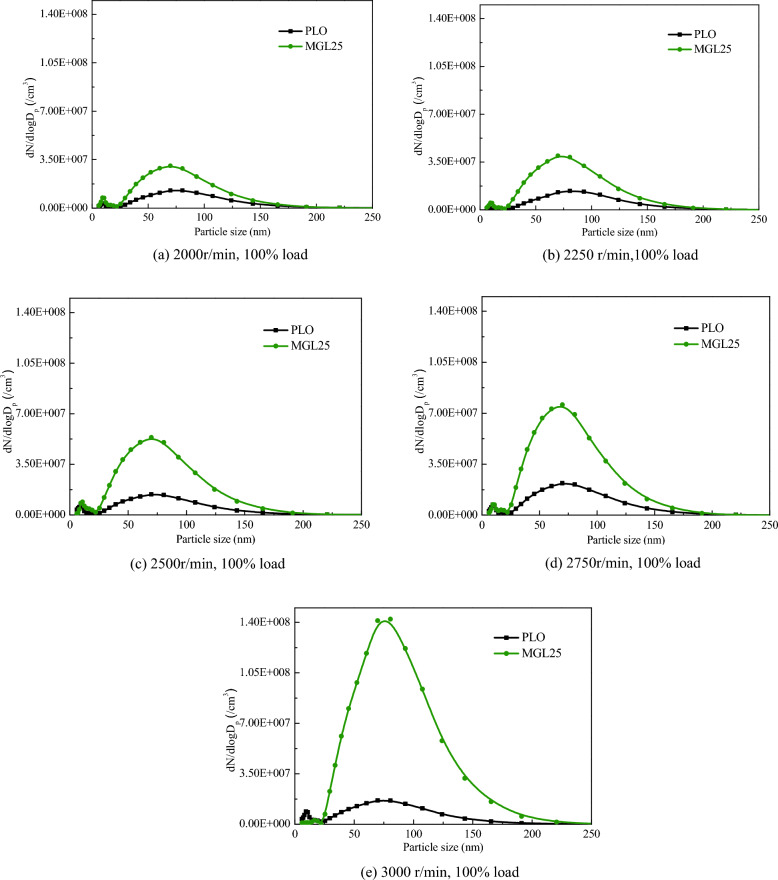
Particle size distribution of different lubricating oils.

Figure 8 shows particle size distribution of different lubricating oils added to the fuel at different speeds at 100% load. It can be clearly seen from the figure that the particle size of 100% load at different speeds presents bimodal logarithmic distribution, with two peaks occurring at 9–19 nm and 69–81 nm respectively. The amount of accumulated particles corresponding to MGL25 is obviously greater than that of PLO, and this phenomenon is more obvious at higher speed.

**Figure 9:**
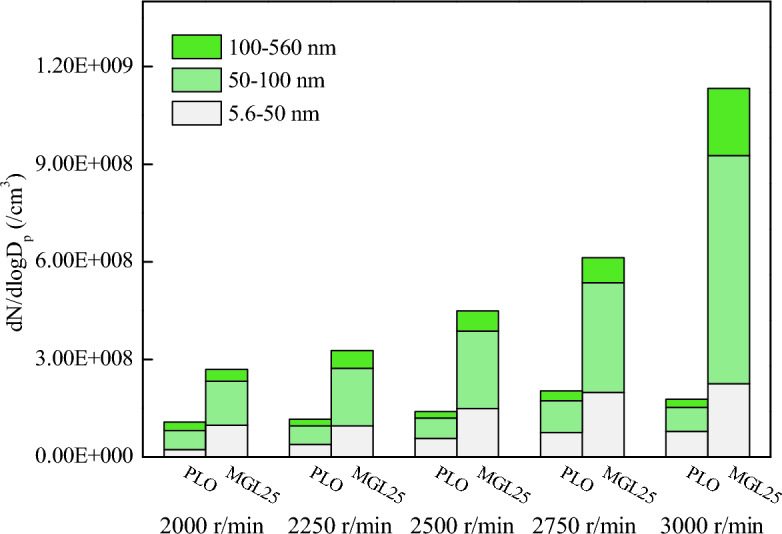
Particle quantity concentration of three particle size intervals corresponding to different lubricating oils at 100% load at different speed.

Particle diameters corresponding to the two lubricating oils under 100% load at different speeds were counted according to three particle size intervals of 5.6–50 nm, 50–100 nm and 100–560 nm. The statistical results are shown in Fig. 9. The particle size corresponding to the two lubricating oils is mostly (75.5–87.6%) concentrated below 100 nm (white and green parts). The number of accumulated particles plays a dominant role in the total number of particles. With the increase of speed, both nuclear particles and accumulated particles increase. This is because with the increase of diesel engine speed, the process of atomization, evaporation and diffusion of fuel injected into the cylinder is shortened, resulting in uneven mixing of fuel and air, thereby increasing PM emissions. The quantity concentration of PM at 5.6–50 nm, 50–100 nm and 100–560 nm corresponding to MGL25 are greater than that of PLO, and the increase in the number of PM in the accumulated state increases more significantly than that in the nuclear state. Therefore, compared with PLO, MGL25 at 100% load at different speeds has more particles, and the phenomenon is more obvious at high speed. The disadvantage of using MGL25 is more prominent. This is due to the existence of nano-graphene, which is more likely to self-nucleate, and more likely to adsorb on the surface of carbon or agglomerate to form larger particle size particles. And the higher the rotational speed, the shorter the combustion duration, the higher the exhaust flow rate, the more likely to agglomerate and produce larger particle size. It is necessary to adjust the control strategy and test and calibrate the post-processing system.

### Microscopic morphology of PM

**Figure 10:**
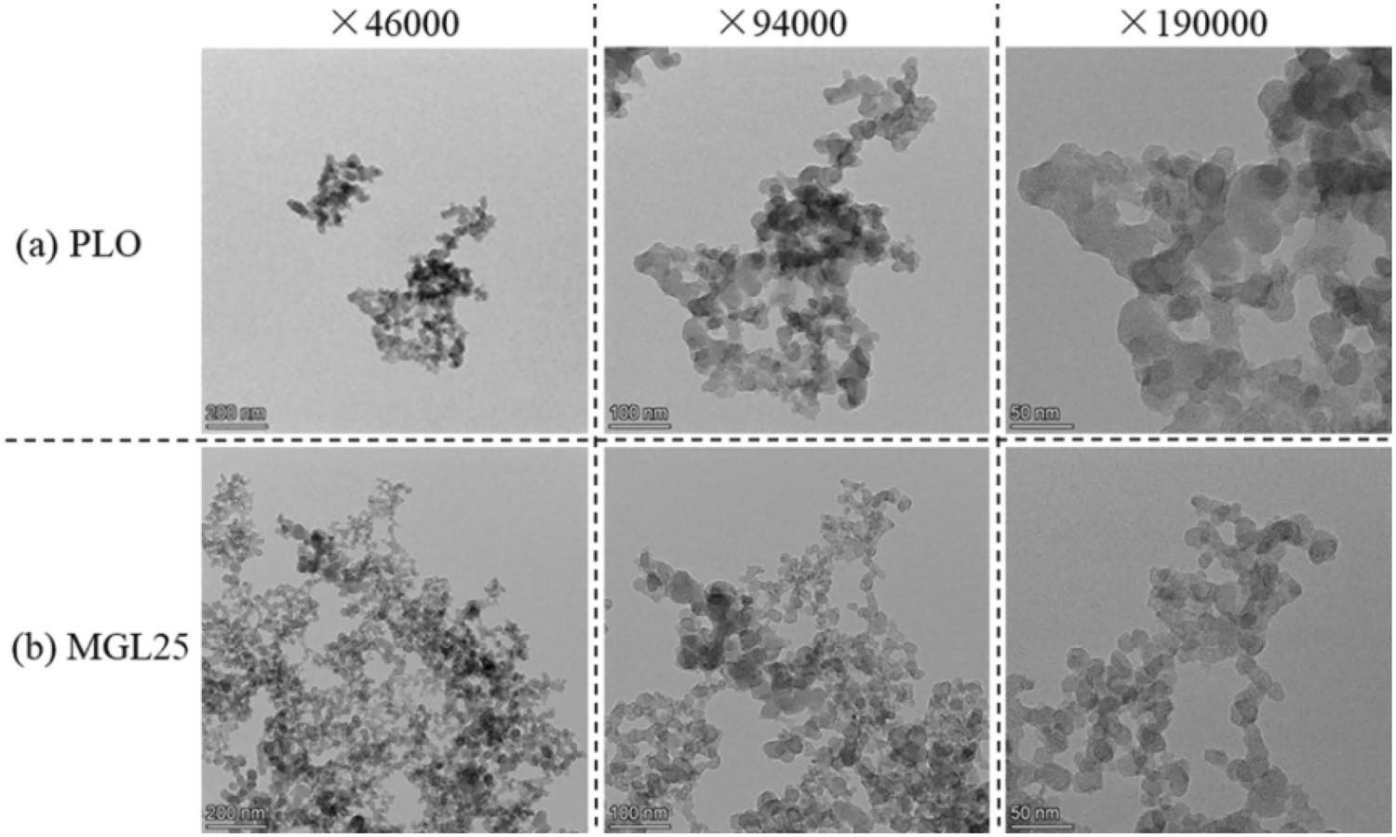
Morphologies of PM at different multiples.

Under rated working conditions, the morphologies of PM corresponding to the two lubricating oils at different multiples (46,000, 94,000 and 190,000 times) are shown in Fig. 10. PM is composed of dozens to hundreds of spheroid basic carbon particles, showing irregular shapes such as clusters, chains, branched and so on. There is no much difference in the intuitive morphology of PM. Nanomaterials do not affect the intuitive morphology of PM. It is worth noting that at 94,000 multiples, it is easy to see that the basic carbon particle size of PM corresponding to PLO is relatively uniform. And the number of basic carbon particles with smaller particle sizes corresponding to MGL25 increased. It is verified that when the nano-graphene lubricating oil participates in combustion, the nano-particles may self-nucleate and produce more and smaller particle size of basic carbon particles.

### Fractal dimension of PM

**Figure 11:**
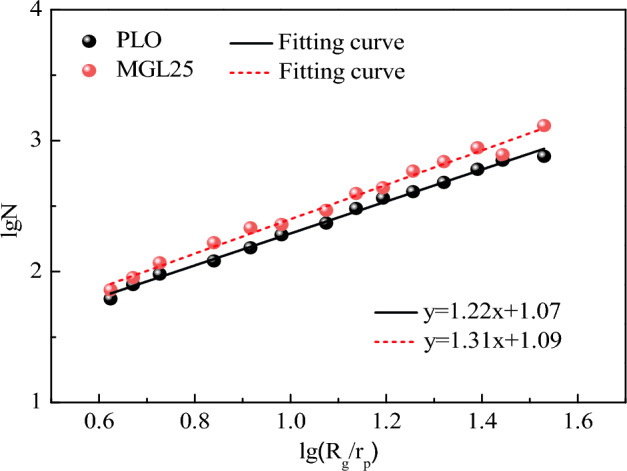
lgN−lg (R_g_/r_p_) scatter fitting diagram of PM.

The lgN−lg(R_g_/r_p_) scatter fitting diagram of PM corresponding to the two lubricating oils is shown in Fig. 11. As can be seen from the figure, the fractal dimension of PM corresponding to PLO and MGL25 are 1.22 and 1.31, respectively. Compared with PLO, the fractal dimension of MGL25 is increased by 7.4%. This indicates that the structure of PM corresponding to MGL25 becomes tighter and more unfavorable to the oxidation of PM.

### Structure of basic carbon particles of PM

**Figure 12:**
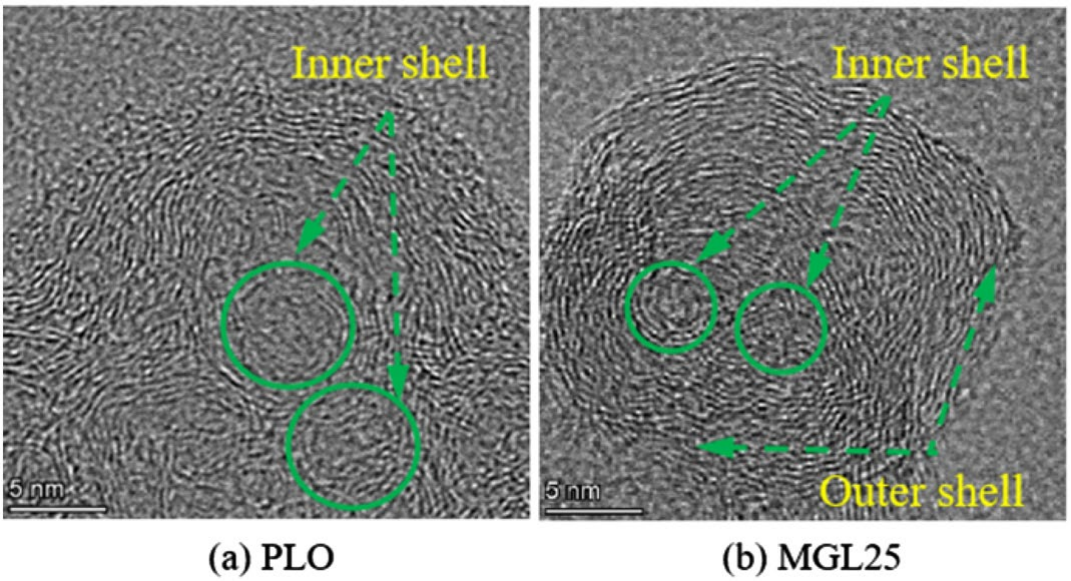
Micromorphologies of the basic carbon particles.

The micromorphologies of the basic carbon particles corresponding to the two lubricating oils are shown in Fig. 12. As can be seen from the figure, the basic carbon particles show a textured spherical carbon layer structure. The microstructure is composed of two parts: the outer shell and the inner core. The outer shell shows distinct and regular microcrystalline carbon layers. The inner core shows one or more vortex spheres, which are caused by the bending, folding and irregular arrangement of the microcrystalline carbon layers. The results agree well with those of previous studies^46,47^.

**Figure 13:**
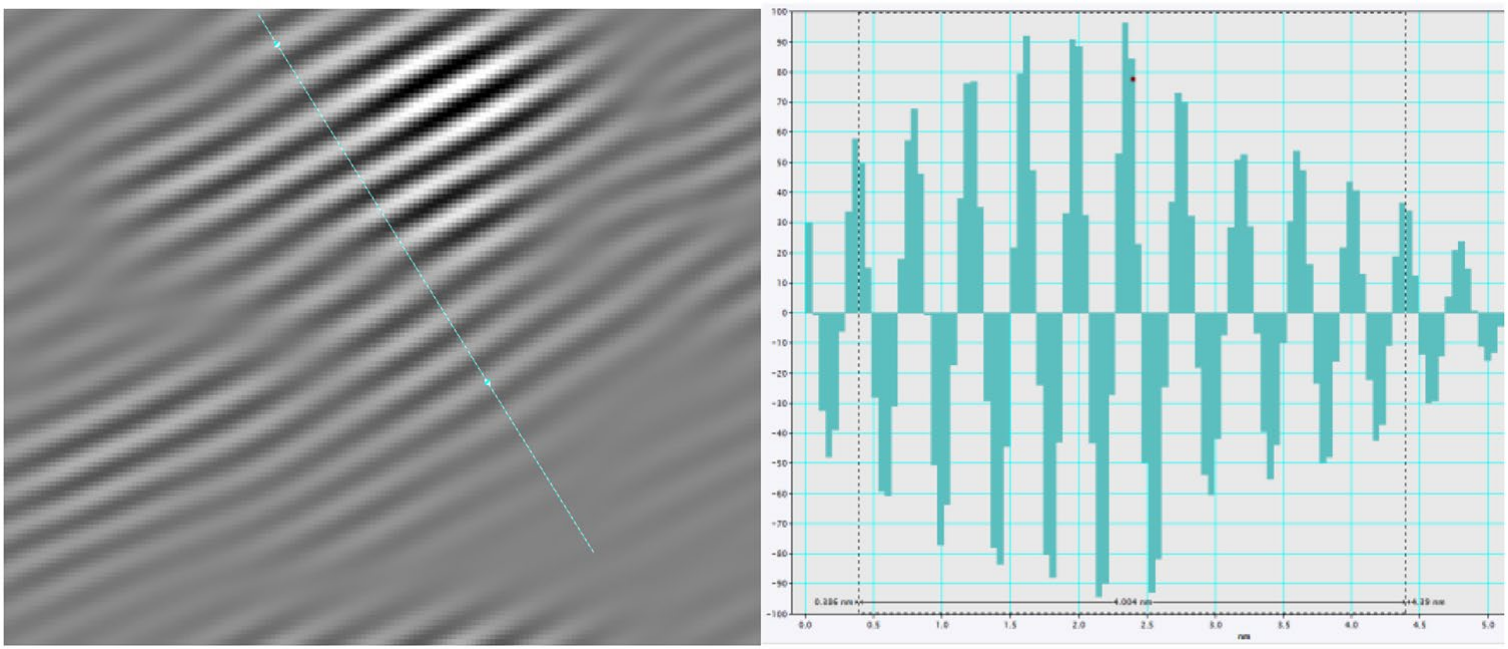
Grayscale distribution of graphite layer.

With the help of Digital Micrograph software, the gray scale distribution of the graphite layer in the vertical direction was obtained by the method of image gray scale measurement. The distance between two adjacent peaks is denoted as the fringe separation distance. In order to reduce the error, 10 fringe separation distance were measured and the average value was taken, denoted as the fringe separation distance of graphite layer, as shown in Fig. 13.

**Figure 14:**
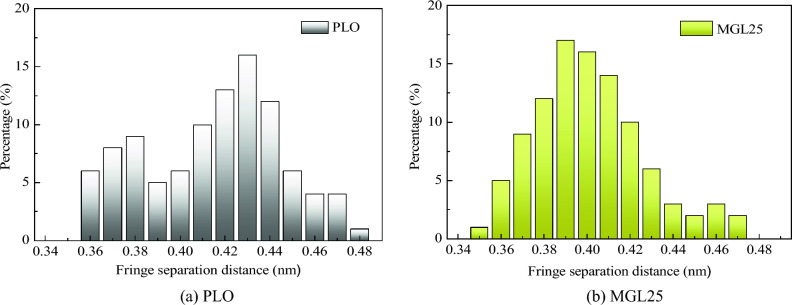
Fringe separation distance distribution of basic carbon particles of PM.

The fringe separation distance distribution of basic carbon particles of PM corresponding to the two lubricating oils is shown in Fig. 14. The fringe separation distance distribution of basic carbon particles of PM corresponding to PLO presents a bimodal curve distribution, and the peak values are concentrated near 0.37 nm and 0.43 nm, respectively. However, after the addition of 25 ppm nano-graphene into the lubricating oil, the peak value near 0.37 nm disappears and the peak value of the curve shifts to the left. It is calculated that the average fringe separation distance of basic carbon particles of PM corresponding to PLO and MGL25 is 0.415 nm and 0.401 nm, respectively. By comparison, it can be seen that the fringe separation distance of PM is reduced by 3.4% after the addition of nano-graphene in lubricating oil, and the possibility of oxygen entering the edge of the layer is less, which is not conducive to the oxidation of PM.

**Figure 15:**
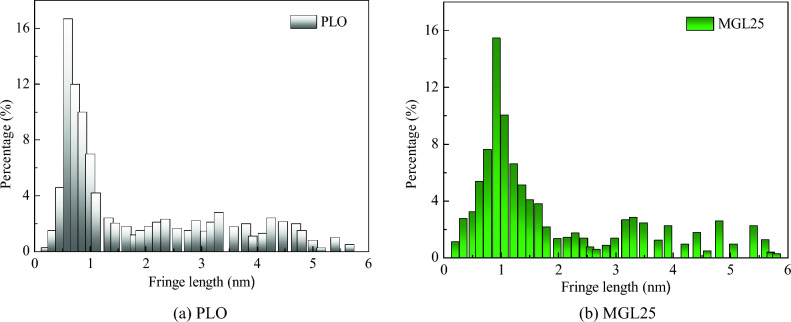
Fringe length distribution of basic carbon particles in two lubricating oils.

The fringe length distribution of basic carbon particles in lubricating oil is shown in Fig. 15. The peak of fringe length distribution of basic carbon particles corresponding to PLO and MGL25 appear near 0.6 nm and 0.92 nm, respectively. Compared with PLO, MGL25 increases the average fringe length of PM by 5.6%. Therefore, compared with PLO, the particles corresponding to MGL25 have higher order degree, higher graphitization degree and lower reactivity, which is not conducive to the oxidation of particles.

**Figure 16:**
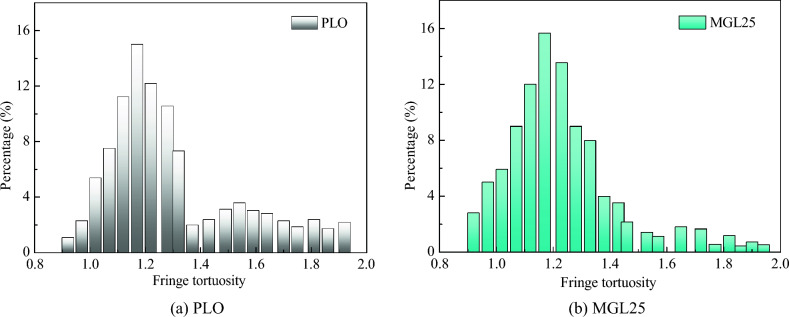
Fringe tortuosity distribution diagram of basic carbon particles.

The fringe tortuosity distribution of basic carbon particles of PM corresponding to two lubricating oil is shown in Fig. 16. The fringe tortuosities are all distributed between 0.8 and 2. The average fringe tortuosity of basic carbon particles of PM corresponding to PLO and MGL25 is 1.30 nm and 1.23 nm, respectively. Compared with PLO, the average fringe tortuosity of basic carbon particles of PM corresponding to MGL25 is reduced by 5.4%. This shows that the tortuosity of basic carbon particles of PM is reduced after the addition of nano-graphene in lubricating oil. It can be seen that the carbon layer structure fluctuation of basic carbon particles of PM after the addition of nano-graphene in lubricating oil is smaller and the structure is more stable.

### Graphitization degree of PM

**Figure 17:**
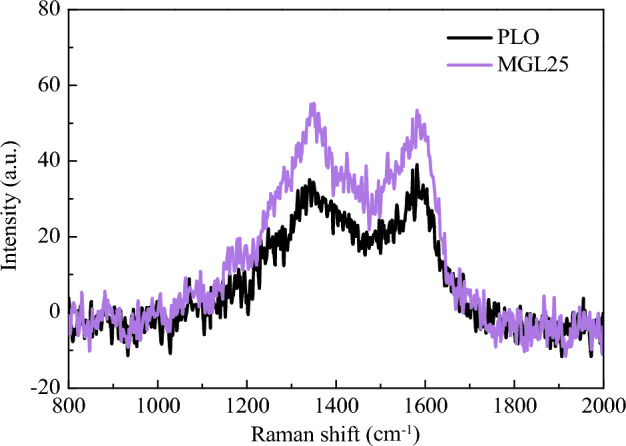
Raman spectrums of PM corresponding to two lubricating oils.

The Raman spectrums of PM corresponding to the two lubricating oils are shown in Fig. 17. It can be clearly seen from the figure that the peak shapes of the spectrums are basically the same, with two characteristic peaks located around 1345 cm^−1^ and 1583 cm^−1^ respectively. This result is consistent with the literature^48^. The characteristic peak near 1345 cm^−1^ is caused by the loss of symmetry of the hexagonal symmetry of the local structure of the crystal or the transformation to a lower symmetry. The PM structure has defects and disordered arrangement at the edges, corresponding to the symmetric vibration of the A_1g_ graphite lattice, which is called D-peak. The characteristic peak near 1583 cm^−1^ is caused by the stretching vibration of sp^2^ hybrid atoms in the carbon layer, which is called G-peak. It can characterize the structure of the ordered carbon layer of PM.

In order to obtain accurate quantification results of graphitization degree of PM, further extraction of Raman spectrogram data is required. The traditional method is to calculate the ratio of peak height of two peaks to characterize the graphitization degree of PM, but this method ignores the influence of some peaks overlap and peak width on the graphitization degree. At present, some researchers have used the four-peak or five-peak method to analyze the graphitization degree of PM. This paper referred to the four-peak fitting method in the reference^49^, and used the Peak Fitting Module function in software origin to fit Raman spectrogram. The four peaks are 1200 cm^−1^ (D4), 1345 cm^−1^ (D1), 1520 cm^−1^ (D3) and 1583 cm^−1^ (G). Among them, the D3 peak was fitted by Gaussian curves and D4, D1 and G peaks were fitted by Lorentz curves. D3 peak is an amorphous carbon type caused by organic molecules and functional groups of PM. D4 is the stretching vibration of carbon bond caused by impurity ions or polyene molecules. Full width at half maximum (FWHM) refers to the difference between the two abscissa coordinates at half of the fitting peak value, indicating the range of chemical action. D1 peak is usually used to fit the FWHM curve to represent the chemical dissimilarity of PM. The narrower the FWHM is, the smaller the substance composition of PM is, and the weaker the chemical dissimilarity is.

**Figure 18:**
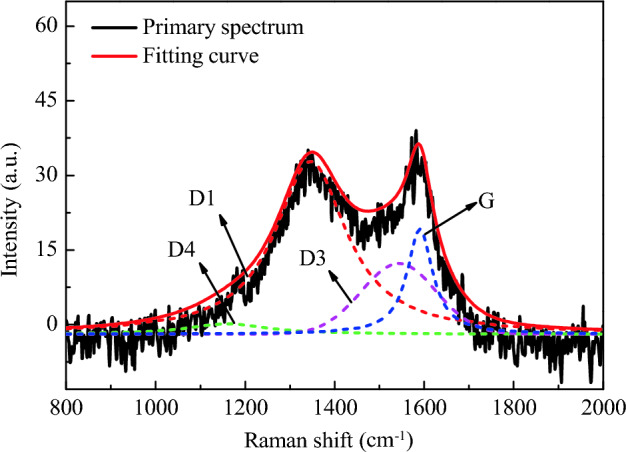
Raman spectrum fitting diagrams of PM corresponding to PLO.

**Figure 19:**
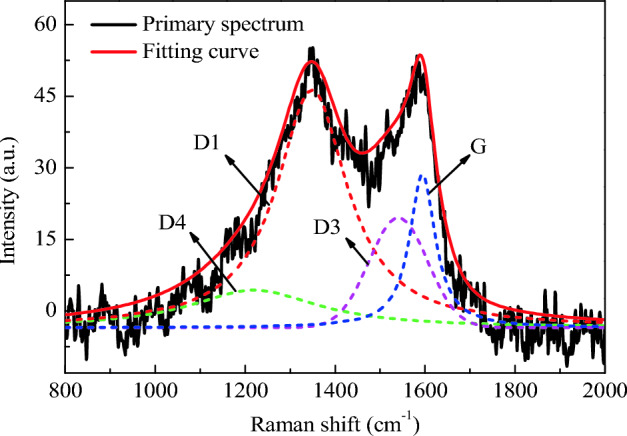
Raman spectrum fitting diagrams of PM corresponding to MGL25.

The Raman spectrum fitting diagrams of PM corresponding to the two lubricating oils are shown in Figs. 18 and 19. The fitting degrees are 96.5% and 97.5% respectively, and the fitting degrees are both higher than 96%, indicating an ideal fitting effect. The position and the FWHM of each peak are shown in Tables 6 and 7. The FWHM of D1 peaks corresponding to PLO and MGL25 are 194.1 and 193.6, respectively. This shows that the chemical heterocorrelation of PM corresponding to the lubricating oil added with nano-graphene is basically unchanged. In this study, the graphitization degree of PM is characterized by the area ratio of D1 peak and G peak, I_D1_/I_G_. The smaller the area ratio is, the higher the graphitization degree is. Conversely, the lower the graphitization degree is. The I_D1_/I_G_ of PM corresponding to PLO and MGL25 is 4.313 and 4.022, respectively. This shows that the graphitization of PM corresponding to the lubricating oil after the addition of nano-graphene increases slightly. This is because MGL25 has higher oxidation characteristics than PLO, resulting in higher graphitization degree of generated PM.

**Table 6:** Peak position and FWHM of Raman spectrum fitting of PM corresponding to PLO.

	D4	D1	D3	G
Peak position (cm^−1^)	1190	1344	1508	1589
FWHM (cm^−1^)	215.0	194.1	197.0	69.0

**Table 7:** Peak position and FWHM of Raman spectrum fitting of PM corresponding to MGL25.

	D4	D1	D3	G
Peak position (cm^−1^)	1210	1349	1531	1590
FWHM (cm^−1^)	349.1	193.6	153.1	69.3

### Surface functional groups of PM

**Figure 20:**
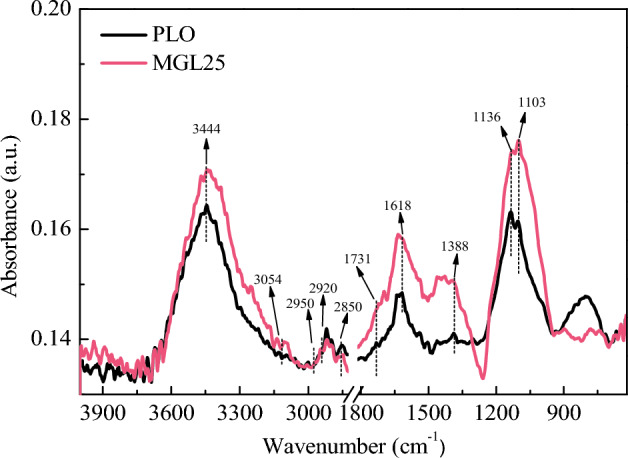
FTIR spectrums of PM corresponding to two lubricating oils.

The FTIR spectrums of PM corresponding to the two lubricating oils are shown in Fig. 20. It can be seen that PM corresponding to the two lubricating oil has similar absorption peak distribution, and the difference is mainly reflected in the peak intensity of the absorption peak. The functional groups of PM mainly include aliphatic functional groups, oxygen-containing functional groups and aromatic functional groups. Among them, three absorption peaks locate near 2950 cm^−1^, 2920 cm^−1^ and 2850 cm^−1^, respectively, correspond to aliphatic C–H groups, methyl and methylene groups mainly from polycyclic aromatic hydrocarbons PAH molecules or PAH inter-molecular bridging. At the same time, due to the deformation of aliphatic C–H groups in the molecular plane, the corresponding absorption peak is generated near 1388 cm^−1^. Oxygen-containing functional groups mainly include C=O groups near 1731 cm^−1^, C–O groups in phenol, alcohol, ether and ester oxygen bonds near 1103 cm^−1^ and 1136 cm^−1^, and OH groups in alcohol, phenol, peroxide, carboxylic acid and water near 3444 cm^−1^. The aromatic functional groups mainly correspond to the C=C group in the aromatic ring or thick ring near 1618 cm^−1^ and the aromatic CH group near 3054 cm^−1^.

There are more aromatic rings, which are not easy to be oxidized, in the PM corresponding to the nano-graphene lubricating oil. In order to avoid the error caused by the different thickness of KBr slices, the absorption peak in the figure is expressed as the ratio of the absolute signal intensity there to the absorption peak intensity at 3444 cm^−1^, that is, the relative absorbance. The absorption intensity values of the main functional groups of PM corresponding to the two lubricating oils are shown in Table 8. 3054 cm^−1^ corresponds to the aromatic –CH stretching vibration peak. 3054 cm^−1^ corresponds to the aromatic CH stretching vibration peak. 618 cm^−1^ corresponds to C=C stretching vibration peak in aromatic ring or thick ring. The characteristic peaks of PM surface aromatic –CH at 3054 cm^−1^ corresponding to the two lubricating oils are not obvious. As can be seen from the absorption intensity value at 3054 cm^−1^, the aromatic –CH composition of the PM corresponding to nano-graphene lubricating oil has little change. However, the bending vibration of the outer plane caused by the single and adjacent hydrogen atoms still exists in the aromatic material, corresponding to the characteristic peak of 867–700 cm^−1^, and the peak value of PM corresponding to the nano-graphene lubricating oil here is smaller than that corresponding to pure lubricating oil. This shows that the content of aromatic –CH of the PM corresponding to MGL25 is reduced. The absorption intensity value at 1618 cm^−1^ shows that the PM corresponding to MGL25 contains more aromatic components. Compared with PLO, the absorbance corresponding to MGL25 at 1618 cm^−1^ increases by 6.04%. 2950 cm^−1^ and 2920 cm^−1^ respectively correspond to the asymmetric stretching vibration peaks of methyl group and methylene group in aliphatic group, and 2850 cm^−1^ corresponds to the symmetric stretching vibration peak of methylene group in aliphatic group, and these three characteristic peaks are obvious. The symmetric methylene stretching vibration at 2850 cm^−1^ and the asymmetric methylene stretching vibration at 2920 cm^−1^ dominate the aliphatic functional groups on the surface of PM. However, the content of unsymmetrical methyl stretching vibration at 2950 cm^−1^ is relatively low. The absorbance at 2920 cm^−1^ is greater than that at 2950 cm^−1^, which indicates that the PM surface contains more methylene functional groups. From the relative absorbance of the two peaks, it can be seen that the aliphatic material of PM corresponding to the nano-graphene lubricating oil has basically no change. The peaks at 1388 cm^−1^ and 1459 cm^−1^ correspond to the symmetric deformation and asymmetric vibration of methyl groups respectively. It can be seen that, compared with PLO, the absorbance of methyl symmetrical deformation vibration peak of the PM corresponding to nano-graphene lubricating oil is greater. This is because aliphatic substances largely replace the active sites of aromatic substances. 1731 cm^−1^ corresponds to the stretching vibration peak of C=O in aliphatic group. 1136 cm^−1^ and 1101 cm^−1^ correspond to phenolic, alcohol, ether and ester oxygen bond peaks respectively. Compared with PLO, the relative absorbances of PM corresponding to nano-graphene lubricating oil increase by 5.04% at 1731 cm^−1^, 6.75% at 1136 cm^−1^ and 9.32% at 1101 cm^−1^. From the point of view of the relative absorbance of the two peaks, compared with PLO, the oxygen-containing functional groups of the PM corresponding to MGL25 increase. In summary, the aliphatic substances in the PM corresponding to MGL25 have little change, the aromatic components and oxygen-containing functional groups increase.

**Table 8:** Absorbance of the main functional groups of PM corresponding to the two lubricating oils.

	OH	Arom-atic	Aliphatic group			C=O in the aliphatic group	Arom-atic ring	Methyl deform-ation	Phenolic, alcohol, ether and ester oxygen bond	
	3444	3054	2950	2920	2850	1731	1618	1388	1136	1103
PLO	0.164	0.136	0.137	0.142	0.139	0.139	0.149	0.141	0.163	0.161
MGL25	0.171	0.136	0.138	0.140	0.137	0.146	0.158	0.151	0.174	0.176

### Oxidation characteristics of PM

**Figure 21:**
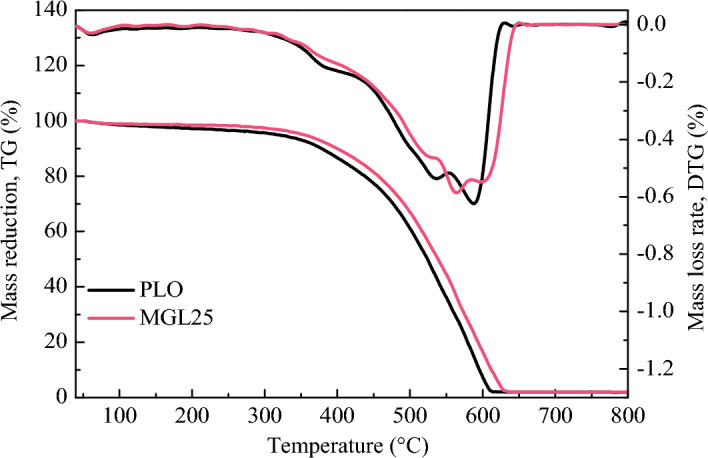
TG and DTG curves of PM corresponding to two lubricating oils.

The TG and DTG curves of PM corresponding to two lubricating oils are shown in Fig. 21. It can be seen from the TG curve in the figure that with the increase of temperature, PM undergo complex physicochemical reactions, including evaporation of water, volatilization of soluble organic fractions (SOF), and pyrolysis of soot. The mass of PM decreases with increasing temperature. When the temperature reaches 650 °C, the PM mass changes very little, indicating that the oxidation process is basically completed. The results are consistent with previous studies^50^. Compared with PLO, the TG curve of the PM corresponding to the lubricating oil added with graphene shifts to the right, that is, to the high-temperature mass loss zone. The mass loss rate decreases slightly in the range of 350–550 °C and increases significantly in the range of 620–670 °C.

The oxidation characteristic parameters of PM corresponding to the two lubricating oils are shown in Table 9. Compared with PLO, the initial oxidation temperature and burnout temperature of PM corresponding to nano-graphene lubricating oil increase, and the maximum oxidation rate temperature and combustion characteristic index decrease.

**Table 9:** PM oxidation characteristic parameters corresponding to two lubricating oils.

Lubricating oil	T_i_ (°C)	T_max_ (°C)	T_h_ (°C)	S (mg^2^·min^−2^·°C^−1^)
PLO	381.3	585.7	596.3	1.20 × 10^–9^
MGL25	403.8	567.7	614.1	0.95 × 10^–9^

**Figure 22:**
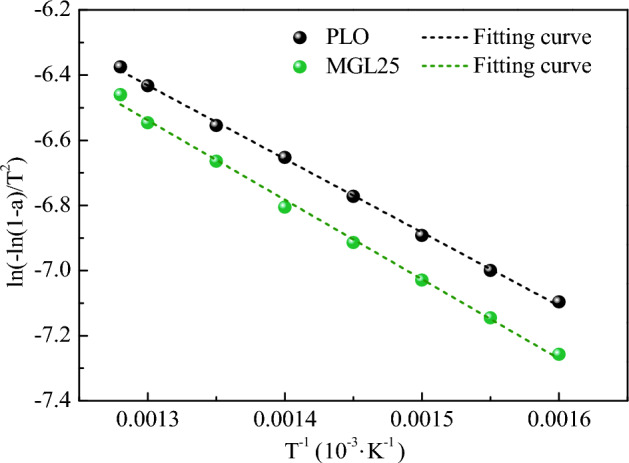
Fitting curves of the relationship between ln[− ln(1−α)/T^2^] and 1/T of PM.

The fitting curves of the relationship between ln[−ln(1−α)/T^2^] and 1/T of PM corresponding to the two lubricating oils are shown in Fig. 22, and the activation energy results are shown in Table 10. The activation energies of PM corresponding to PLO and MGL25 were 18.76 kJ/mol and 20.29 kJ/mol, respectively. Compared with PLO, the activation energy of PM corresponding to MGL25 increases by 8.16%. PM corresponding to MGL25 is more difficult to oxidize, which is mainly due to the higher degree of graphitization of PM corresponding to MGL25 and the increased content of aromatic substances.

**Table 10:** PM activation energy corresponding to two lubricating oils.

Lubricating oil	Fitting curve equation	Slope	Activation energy (kJ/mol)
PLO	y = − 2256.41x−3.50	− 2256.41	18.76
MGL25	y = − 2440.15x−3.37	− 2440.15	20.29

## Conclusions

EEPS was used to measure particle size distribution corresponding to diesel with different lubricating oils under different working conditions. In addition, a self-made PM sampling device was used to collect PM at the rated working condition, and the physicochemical properties of PM were analyzed by comparing and studying its microscopic morphology, structure, surface functional groups and oxidation properties. The main conclusions are as follows:

1. Under different working conditions, particle sizes corresponding to the two lubricating oils are mostly concentrated below 100 nm. The quantity concentration of nuclear particles, accumulated particles and total particles corresponding to MGL25 are significantly higher than that corresponding to pure lubricating oil, and the increase in the number of accumulated particles is more obvious than that of nuclear particles. And this phenomenon is more obvious when the load is larger and the speed is larger. Nano-graphene lubricating oil has adverse effects on the post-processing system, and the disadvantage of using nano-graphene lubricating oil is more prominent. It needs to adjust the control strategy and test and calibrate the post-processing system of the engine. And there is a risk of atmospheric environment pollution.
2. There is no much difference in the intuitive morphology of PM corresponding to PLO and MGL25. The basic carbon particles of PM corresponding to PLO have uniform particle size, while the number of basic carbon particles with smaller particle size of PM corresponding to nano-graphene lubricating oil increases. It confirms that nano-particles will nucleate themselves when nano-graphene lubricating oil is involved in combustion, increasing the risk of generating more nuclear particles.
3. Compared with PLO, the fractal dimension of the PM corresponding to MGL25 is larger and the structure is more compact. The average PM fringe separation distance of MGL25 decreases, the average fringe length increases, and the degree of PM ordering and graphitization are higher. The fringe tortuosity of PM basic carbon particles of MGL25 decreases, and the fluctuation of carbon layer structure of basic carbon particles decreases.
4. After the addition of nano-graphene in lubricating oil, the aliphatic substances in the generated PM are basically unchanged, but the aromatic components are increased, and the oxygen-containing functional groups in the PM are increased.
5. Compared with PLO, the initial PM oxidation temperature and burnout temperature corresponding to MGL25 increase, while the maximum oxidation rate temperature and combustion characteristic index decrease. The activation energies of PM corresponding to PLO and MGL25 are 18.76 kJ/mol and 20.29 kJ/mol, respectively. This indicates that the PM corresponding to MGL25 is more difficult to oxidize. This is mainly due to the higher degree of graphitization of the PM corresponding to MGL25, and the increased content of aromatic substances.

## Data Availability

Datasets will be available upon request, contact Xiping Yang at: yxp@jssc.edu.cn.
